# Necrotizing Fasciitis Secondary to* Aeromonas* Infection Presenting with Septic Shock

**DOI:** 10.1155/2017/4607582

**Published:** 2017-08-31

**Authors:** Nikhil Bhatia, Manuel Castro-Borobio, John N. Greene, Sowmya Nanjappa

**Affiliations:** ^1^University of South Florida Morsani College of Medicine, 12901 Bruce B. Downs Blvd, Tampa, FL 33612, USA; ^2^Department of Infectious Disease, University of South Florida Morsani College of Medicine, 12901 Bruce B. Downs Blvd, Tampa, FL 33612, USA; ^3^H. Lee Moffitt Cancer Center and Research Institute, 12902 Magnolia Drive, FOB-3, Tampa, FL 33612, USA; ^4^Department of Internal Medicine and Department of Oncologic Sciences, H. Lee Moffitt Cancer Center and Research Institute, 12902 Magnolia Drive, Tampa, FL 33612, USA

## Abstract

This report describes a case of necrotizing fasciitis presenting with septic shock due to an* Aeromonas* infection. The patient cut his foot while mowing the lawn and then spent time in a pool with black mold. He began feeling ill and developed swelling and a quarter-sized black area on his right lower extremity. Despite being hemodynamically unstable with systolic blood pressure in the low 70s, the patient was transferred to our facility from outside hospital 100 miles away. Upon arriving to facility, the patient appeared to be septic and the infected area of skin had grown. Irrigation and debridement were performed and appropriate antibiotic therapy was given; however, the patient subsequently died on hospital day 8. On review of the literature, cases of necrotizing fasciitis due to* Aeromonas* infection have been treated successfully with the aforementioned therapy; however, there is high mortality associated with these infections, many times related to a delayed diagnosis. Our patient also had multiple poor prognostic factors including hepatic dysfunction and immunosuppression.

## 1. Introduction

The genus* Aeromonas* is characterized by Gram-negative bacilli that are facultative anaerobes. The bacteria are usually found in fresh water, sewage, and soil.* Aeromonas* are known to cause human infections. The most common infections caused by* Aeromonas* are gastrointestinal and skin or soft tissue wound infections; however, it is also known to cause pneumonia, meningitis, osteomyelitis, endocarditis, and septic arthritis. Necrotizing fasciitis is a serious infection of the superficial fascia. It is most commonly found in patients with hepatic disease, diabetes, or immunocompromised status. We report a case of* Aeromonas* necrotizing fasciitis with progression to septic shock by a patient who had the following risk factors: metastatic cancer, recent corticosteroid use, trauma causing break in the skin, and recently being in a pool with mold and delayed treatment.

## 2. Case Presentation

The patient is a 60-year-old male with a history of metastatic lung cancer to the lung, liver, and brain. The patient presented to an outside hospital's ER due to rapidly progressive right foot pain, swelling, and a black area on right lower extremity. About a week earlier, he had scratched the anterolateral part of his ankle while mowing his lawn. He subsequently worked on his pool which was known to be dirty with black mold. The patient was found to have a quarter-sized lesion on right lower extremity. Then he began feeling more sick and went to a local hospital. That same night, the patient was transferred to Moffitt Cancer Center from over 100 miles away, despite his blood pressure being in the 70s. On admission, the patient appeared to be in sepsis and was immediately transferred to ICU to start hemodynamic support. The patient's past medical history includes a progressive cancer despite frontline chemotherapy that he last received 2 months earlier. He was to be enrolled in a clinical trial when screening evaluation found a new brain metastasis and had since received whole-brain radiation therapy. Medications included dexamethasone 4 mg twice a day for the past 3 weeks and Xarelto for history of pulmonary embolus.

The patient was afebrile with a temperature of 97.8 F. Pulse was 89 and respiratory rate was 23. Of note, the patient was hypotensive with a blood pressure of 72/50. He was alert and oriented and in considerable distress. On physical exam, the patient was found to have warm extremities with bilateral pitting edema. His right foot was erythematous and tender on palpation. His wound had gotten worse as there was purplish discoloration in a 10 cm area with possible necrosis of skin. No crepitus was felt. Also, the patient had thrush and no lymphadenopathy with no other skin lesions noted. Lab results showed the following: negative urine nitrite and leukocyte esterase, WBC of 18.23 k/ul, neutro auto of 15.24 k/ul, prothrombin time of 17.9 s, APTT of 35.3 s, INR of 1.9, glucose of 47 mg/dl, BUN of 32 mg/dl, creatinine of 1.7 mg/dl, estimated GFR of 41 ml/min/1.73 m^2^, albumin of 2.5, total bilirubin of 4.9 mg/dl, alkaline phosphatase of 182 U/L, AST of 173 U/L, ALT of 130 U/L, and lactic acid of 4.8 mmol/L. His initial ABG after intubation showed pH of 7.08, pCO_2_ of 54, and pO_2_ of 62 on 100% O_2_. His chest X-ray showed what appeared to be acute respiratory distress syndrome.

During initial resuscitation, the patient was treated with maximum levophed dose and systolic blood pressure in the low 100s was obtained. He then developed atrial flutter with heart rate in the 200s. After giving 5 mg of metoprolol and holding levophed, the patient converted to normal sinus rhythm. Systolic blood pressure again dropped into the 50s and the patient was again given levophed along with phenylephrine and vasopressin and blood pressure rose to the 70s. He needed to be intubated due to progressive hypoxia. The patient had an episode of aspiration with a 10-second period of O_2_ dropping to 30% during procedure. He eventually had successful intubation.

For empiric coverage, the patient received vancomycin, meropenem, and doxycycline. A diagnosis of septic shock with multiple organ failure secondary to necrotizing fasciitis was made and urgent surgery consult was placed. The patient was also found to be coagulopathic, in acute renal failure, acidotic, and in acute respiratory failure. Surgery agreed with the diagnosis and the patient was emergently sent to OR for irrigation and debridement, about 2 to 3 hours after transfer to our institution. In the OR, purulent material was found coming out from the fascia. The material was cultured. Full thickness skin necrosis was found throughout most of the anterolateral aspect of the leg. The entire skin was removed in excess of a 15 by 20 cm area with 1 cm of subcutaneous tissue. Thorough irrigation was done with over 6 liters of normal saline with bacitracin in two different compartments of the leg. Despite the extensive infection, the muscles appeared to be healthy. At the end of the procedure, the patient was stable and ultimately required less pressor drugs.

Due to extensive coagulopathy, it was not deemed safe for the patient to go back to the OR, so surgery was unable to reevaluate wound the next day as planned. After correcting his coagulopathy, he was taken back to the OR on hospital day 4 for elevated lactic acid and possible ischemic bowel. During the procedure, the bowel appeared to be healthy; however, the liver was found to be replaced by tumor and physiologically the patient appeared cirrhotic. Wound cultures taken during initial debridement were found to grow nonlactose fermenting, oxidase-positive, Gram-negative rods. Cultures were later confirmed to grow* Aeromonas hydrophila* and* Aeromonas caviae*. The cultures showed sensitivity to ceftriaxone, ciprofloxacin, gentamicin, tobramycin, and piperacillin/tazobactam.

Despite treatments, on hospital day 8, the patient developed severe subcutaneous emphysema with unclear cause. After discussion with the patient's family, the decision was made to perform terminal extubation, given the patient's underlying stage IV lung cancer and worsening clinical status.

## 3. Discussion

*Aeromonas* species are part of the Aeromonadaceae family and are increasingly associated with human infections. The second most common site of infection caused by* Aeromonas* is the soft tissue underlying the epidermis. The infections associated with* Aeromonas* can range from mild infections to serious ones. These life-threatening infections have varied presentations from cellulitis, necrotizing fasciitis, and myonecrosis.

*Aeromonas* are commonly found to replicate in water. Our patient's exposure to a dirty pool facilitated a higher risk of soft tissue infection by* Aeromonas*. According to Voss, 82% of all* Aeromonas* wound infections resulted from a penetrating injury and 43% of these infections were water-related [1]. These infections commonly occur, as it did with our patient, due to occupational or recreational water-related activity. Our patient's infection was located in the lower extremity which, according to Janda and Abbott, is the most common site of* Aeromonas* infection [2]. Along with exposure to water,* Aeromonas* infection has also been associated with exposure to soil-contaminated objects [3]. The penetrating trauma from mowing the lawn and subsequent activity in a dirty pool most likely exposed our patient to* Aeromonas*.

Based upon data collected in 1998, the overall incidence of* Aeromonas* infections was 10.6 per 1 million people, with wound infections present in 0.7 per 1 million people. One can assume that the amount of wound infection progressing to septicemia would be even lower than 0.7 per million population [4]. It has been found that certain patient characteristics predict a higher risk of developing septic shock. Janda and Abbott [2] proposed four groups of* Aeromonas* septicemia disease presentations. Each group has its own typical patient characteristics, underlying risk factors, precipitating events, and mortality. One of the most common groups to acquire* Aeromonas* septicemia is immunocompromised individuals. The usual precipitating events for being immunocompromised are recent chemotherapy and neutropenia. The most common underlying risk factors are hepatobiliary disease and malignancy. In this group, one common method of entry is through soft tissue and the associated mortality is 32–45% [5]. Another group consists of those that develop sepsis as a result of severe wound infections due to penetrating injuries. Our patient was exposed to both a penetrating trauma and dirty water before developing a severe wound infection. He may also have been immunosuppressed due to his recent chemotherapy 2 months earlier. He had underlying risk factors such as hepatobiliary disease and malignancy. Together, these factors led to septic shock secondary to necrotizing fasciitis.

To decrease morbidity and mortality associated with these infections, prompt clinical diagnosis and aggressive irrigation and debridement are essential. In this particular case, the diagnosis was delayed due to the time spent transferring the patient from the local hospital, more than 100 miles away, to our hospital. According to Wong and Wang, the diagnosis of necrotizing fasciitis is commonly delayed. In an analysis of 89 patients with necrotizing fasciitis, they found that only 15% were diagnosed with or suspected of having necrotizing fasciitis, while 58% of patients were actually diagnosed with cellulitis [6]. Park describes a case of necrotizing fasciitis caused by an* Aeromonas* infection that also had a delayed diagnosis. Like our case, the delayed diagnosis and treatment resulted in the patient developing shock with multiple organ failure [7]. One possible cause of delayed diagnosis is that initial presentation of necrotizing fasciitis is very similar to cellulitis. Early signs that may indicate an infection from* Aeromonas* are rapid onset of cellulitis within 72 hours after an injury that is characterized by pain, swelling, hemorrhagic bullae, subcutaneous bleeding, purpura, necrosis, or gangrene [8].

While clinical isolates of* Aeromonas* have been found to have wide ranging antibiotic sensitivity, they are almost all resistant to penicillin, ampicillin, carbenicillin, and cefazolin [5]. According to Tsai et al., it has been suggested that empiric coverage for* Aeromonas* infection should be started with ceftazidime, cefepime, amikacin, aztreonam, imipenem, or fluoroquinolones [9]. There have also been emerging reports of growing resistance to antibiotics due to expression of different classes of beta-lactamases [8]. After testing cultures for antibiotic susceptibility, we determined that ciprofloxacin, ceftriaxone, tobramycin, and gentamycin could all be effective against the* Aeromonas* infection. Our patient was given a regimen that included meropenem, clindamycin, and doxycycline. It is extremely important to have early surgical irrigation and debridement along with appropriate antibiotic coverage for optimal management.

There have been cases where necrotizing fasciitis due to* Aeromonas* infection has been successfully treated; however, our case was complicated by delayed treatment and multiple risk factors associated with a poor prognosis. Necrotizing fasciitis is more commonly found in patients with liver disease or malignancy [8]. Not only did our patient have a chronic malignant condition in lung cancer but also he had metastatic spread to the brain, lungs, and liver. The liver was found to be almost completely replaced by tumor upon surgical exploration. As discussed previously, hepatic dysfunction is an underlying risk factor for septic shock in* Aeromonas* infection. The liver is responsible for the production of thrombopoietin, a hormone that helps regulate platelet production in bone marrow [10]. Platelets are involved in the release of cytokines that mediate inflammatory responses. Hepatic damage could decrease lymphocyte and complement activation involved in the immune response to bacteria. Liver damage also interferes with the renin-angiotensin system which would be important to compensate for the hypotension associated with septic shock. Our patient needed to be resuscitated with fluids and pressor drugs multiple times. In conclusion, delayed therapy and multiple poor prognostic factors such as hepatic dysfunction, malignancy, and immunodeficiency contributed to our patient's unsuccessful clinical course. For patients with necrotizing fasciitis along with underlying chronic illnesses, urgent surgical debridement is needed due to the severity of the infection.

Early diagnosis and treatment of necrotizing fasciitis are critical. One useful diagnostic tool can be the Laboratory Risk Indicator for Necrotizing Fasciitis (LRINEC), but the diagnostic aids for this entity are limited. The lack of clinical suspicion and clear diagnosis ultimately delays therapy and increases the overall mortality.
